# Horrors and Abuses of the Old Madhouses

**Published:** 1918-08-24

**Authors:** 


### PAPERS ON MADNESS.

### II.—The Horrors and Abuses of the Old Madhouses.

There is nothing nowadays repellent or forbidding about the external aspect or the internal arrangement of a lunatic asylum. In the latter half of the last century, philanthropy was very active, and the managers of lunatic asylums vied with one another in improving the treatment of the mad. No rational medical treatment was then known—very little is known now—and the enthusiasm that in other departments of medicine and in surgery expended itself upon research and upon new methods of treatment founded upon research, was in asylums lavished upon increasing the comfort of the material surroundings of the patients. If they could not be cured, they should at least be comfortable, such was the laudable determination of the managers.

### Treatment by Bricks and Mortar.

Consequently, it seemed at one time as if the intention was to treat madness by bricks and mortar, so palatial were the buildings erected to accommodate the insane poor; and the buildings selected for conversion into private asylums were the abandoned mansions of impoverished noblemen, or even the vacated abodes of Royalty itself. The Commissioners in Lunacy, in their periodical visits of inspection, if they could not report startling discoveries in the pathology and treatment of the disease, were careful to note the decoration of the wards by pictures, flowers, and canary birds. The ingenuity of the medical staff was expended upon devising forms of fireguards, window-bars, gas-brackets, baths, water-closets, and so forth, that should give the greatest security against attempts at self-injury, and at the same time should be as little suggestive of the asylum as possible.

### The Persistence of Tradition.

Nevertheless, in spite of pictures, flowers, and canary birds, in spite of weekly dances and occasional theatrical performances, in spite of cricket matches and garden parties, much of the old horror and repulsion clung, and still clings, about the lunatic asylums, so true it is that the evil that men do lives after them. It is now more than seventy years, more than two generations, since the gross abuses of the old madhouses were swept away and abolished; but the tradition remains, and no doubt will remain for long years to come. When the occasional tourist visits some ancient church in a remote country village, the ancient sexton who conducts him over the building will show him the broken noses of the recumbent figures of departed local magnates, the empty niches in which statues once stood, the plain glass that replaces the glorious colours of the mediæval window, and will explain that all this damage was done in former times by the orders of Cromwell; and if the sexton has a smattering of history he will particularise, and say by the soldiers of Cromwell, and may even show their bullet marks on the church walls and doors. The sexton is right, and he is wrong. He is repeating a tradition that is tenacious, but that is become distorted in the lapse of centuries, and in the transmission from mouth to mouth, and from generation to generation. The damage is attributable to Cromwell, but not to the great Lord Protector to whom our thoughts naturally revert. It is due to a previous bearer of the name, the great Thomas Cromwell, Earl of Essex, the Minister of Henry VIII., and the heavy *malleus maleficorum* of the sixteenth century. The tradition is perverted, but it persists. It has persisted for ten generations, and no doubt will persist for many more. And so it is with the tradition of the cruelty and barbarity of the madhouses. It is but two generations since they were swept away, but the cruelty and barbarity were so extreme and revolting that the tradition of them still clings about the asylums of the present day, and ignorant people like Mr. Bernard Shaw can still be found to declare that the managers of asylums send out press-gangs to waylay and capture sane persons who are inconvenient to their relatives, to hurry them into lunatic asylums, which for the nonce are denominated "charnel houses," and there to torture them to death.

### Horrible Atrocities.

A tradition so tenacious must have a strong foundation. It must be founded upon occurrences that, though now forgotten, or, if remembered, are remembered in confusion and error, as the ecclesiastical depredations of Cromwell are remembered; but it must be founded upon facts that made a very strong impression, and roused very strong feelings at the time they occurred; and such facts there were in abundance. In 1814 a Committee of the House of Commons sat to investigate the abuses then alleged to exist in madhouses, and amongst other evidence was the following: A young woman, who "though requiring some restraint was perfectly harmless," was found chained to the ground by both legs and both arms. In the asylum at York persistent efforts at length discovered four cells that had been sedulously concealed from the committee of investigation. The state of these cells was "dreadful beyond description." One was about eight and a half feet square, perfectly dark, and the stench was almost intolerable. It was occupied at night by three or four women. The thirteen women who occupied these cells at night were in the day-time kept in a room twelve feet by eight, with a window that did not open. The patients in Bethlem were all, at certain times of the year, "physicked, bled, bathed, and vomited" once a week for a certain number of weeks. In the same institution, eight, ten, or more of the patients were chained to the tables in a state of perfect

☛ The previous article appeared on March 2, page 455.

**PAPERS ON MADNESS**—*(continued).*

nudity. One woman had been thus chained for eight years. In another asylum a woman was kept in a pig-sty, ironed in a crib, with wrist-locks and leg-locks, and a chain two or three times across her body; and when she was allowed to walk about, an iron bar was chained to her legs to prevent her from escaping. It was chained to each ankle, and another chain brought up between her legs was attached to her handcuffs. While chained down in the crib, she was occasionally lashed with a horse-whip until the blood followed the strokes. In another madhouse was found, in 1820, an outhouse, ventilated only by cracks in the wall. The door was padlocked, and when it was opened three women were found in the outhouse, one of whom was chained by the arms, wrists, and legs, and by chains round her body, to a crib, in which she lay on the bare rails, without mattress or straw intervening. She was covered only by a rug. In another place the patients were chained in their cribs, wallowing in their own filth, from Saturday to Monday morning, when they were taken out in a state of nudity, covered with sores and ordure, and carried into the yard to be suddenly drenched in cold water, even when ice was in the pails. *This house was good as compared with others, if not much better than many of them.*

What Lord Shaftesbury Found.

Lord Shaftesbury, for many years chairman of the Lunacy Commission, described what he found when the Commission was first constituted in 1828. "One of the first rooms we went into contained nearly one hundred and fifty patients, in every form of madness, a large proportion of them chained to the wall, some melancholy, some ferocious, but the noise and din and roar were such that we positively could not hear each other. . . . Turning from that room, we went into a court appropriated to the women. In that court there were from fifteen to twenty women, whose sole dress consisted of a piece of red cloth, tied round the waist with a rope . . . they were crouching on their knees, and that was the only place where they could be. I do not think I ever witnessed brute beasts in such a condition, and this had subsisted for years, and no remedy could be applied to it."

Two Lord Chancellors' Responsibility.

Again and again the matter was investigated by Committees of the House of Commons. Again and again Bills were framed, passed through the House of Commons, and sent up to the Lords; and again and again these Bills were rejected owing to the paramount influence exerted over the House by the Lord Chancellor; first Lord Eldon, and after him Lord Brougham.

Eldon was a Tory of the Tories, the type and exemplar of the obstructive obscurantist Tory, who considered that all was for the best in the best possible of worlds and of countries, and that to change anything that had been transmitted to his age by the wisdom of previous generations was sacrilege and abomination. He devoted the whole of a very long and distinguished and laborious life—he was Lord Chancellor for twenty years together—to delaying and obstructing the course of justice in the Court of Chancery, and to delaying and obstructing the course of legislation in the Upper Chamber. Few men, probably, have done more harm in their generation, and few men have received from the State he served so ill more ample and abundant reward. He was successful in preventing for the whole term of his Chancellorship any amelioration of the lot of the insane.

"An Abominable Piece of Legislation."

Eldon was followed by Brougham, the Tory of Tories by the Whig of Whigs. Brougham was the champion of Reform, of Progress, of Liberty. He was regarded in his day by the Tory party with all, and more than all, the hatred and distrust with which Gladstone was regarded by the same party in the next generation. He was looked upon as a man who, for the sake of Reform, and to satisfy his insatiable ambition to have his name associated for ever with Reform, would subvert the Crown and wreck that idol of the time, the great British Constitution; and what was Brougham's attitude towards the Bills that attempted to put a stop to the horrible atrocities that have been described above? It shall be given in his own words. "This," he said, "is one of the most abominable pieces of legislation that ever was seen." "It is monstrous." "Their lordships could never suffer such an abominable piece of legislation to be thrust down their throats."

Years of Delay and Obstruction.

Between them the two Lord Chancellors, the Tory and the Whig, succeeded in perpetuating for many years the horrible state of things we have described. At that time public opinion had very little influence upon the course of legislation. The House of Commons was the close preserve of the two oligarchies of the Tories and the Whigs. The press had little influence. Nothing was done. And throughout all those years of delay and obstruction the horror and hatred of madhouses deepened and festered in the public mind. Is it to be wondered at that a sentiment so deep-rooted, so powerful, so long in growth, should endure for long, should continue active for many years after the state of things that amply justified it has long passed away, and should even now invest the palatial abodes in which the insane are housed in comfort, and even in luxury, with something of the same horror and loathing that attached to the old madhouses?

*(To be continued.)*

